# High genetic diversity but no geographical structure of *Aedes albopictus* populations in Réunion Island

**DOI:** 10.1186/s13071-019-3840-x

**Published:** 2019-12-19

**Authors:** Anne C. Latreille, Pascal Milesi, Hélène Magalon, Patrick Mavingui, Célestine M. Atyame

**Affiliations:** 1 Université de La Réunion, UMR PIMIT “Processus Infectieux en Milieu Insulaire Tropical”, INSERM U1187, CNRS 9192, IRD 249, Plateforme de Recherche CYROI, Saint Clotilde, La Réunion, France; 2 Department of Ecology and Genetics, Evolutionary Biology Center, Science for Life Laboratory, Uppsala University, Uppsala, Sweden; 3 Université de La Réunion, UMR ENTROPIE “Ecologie Marine Tropicale des Océans Pacifique et Indien”, CNRS-IRD-Université de La Réunion, La Réunion, France

**Keywords:** Asian tiger mosquito, Population genetics, Microsatellite, Gene flow, Réunion Island

## Abstract

**Background:**

In recent years, the Asian tiger mosquito *Aedes albopictus* has emerged as a species of major medical concern following its global expansion and involvement in many arbovirus outbreaks. On Réunion Island, *Ae. albopictus* was responsible for a large chikungunya outbreak in 2005–2006 and more recently an epidemic of dengue which began at the end of 2017 and is still ongoing at the time of writing. This dengue epidemic has seen a high number of human cases in south and west coastal regions, while few cases have been reported in the north and east of the island. To better understand the role of mosquito populations in such spatial patterns of dengue virus transmission in Réunion Island, we examined the genetic diversity and population structure of *Ae. albopictus* sampled across the island.

**Results:**

Between November 2016 and March 2017, a total of 564 mosquitoes were collected from 19 locations in three main climatic regions (West, East and Center) of Réunion Island and were genotyped using 16 microsatellite loci. A high genetic diversity was observed with 2–15 alleles per locus and the average number of alleles per population varying between 4.70–5.90. Almost all *F*_*IS*_ values were significantly positive and correlated to individual relatedness within populations using a hierarchical clustering approach based on principal components analyses (HCPC). However, the largest part of genetic variance was among individuals within populations (97%) while only 3% of genetic variance was observed among populations within regions. Therefore, no distinguishable population structure or isolation by distance was evidenced, suggesting high rates of gene flow at the island scale.

**Conclusions:**

Our results show high genetic diversity but no genetic structure of *Ae. albopictus* populations in Réunion Island thus reflecting frequent movements of mosquitoes between populations probably due to human activity. These data should help in the understanding of *Ae. albopictus* vector capacity and the design of effective mosquito control strategies.

## Background

The Asian tiger mosquito, *Aedes* (*Stegomyia*) *albopictus* [1], originated from Southeast Asia [2] is currently among the most invasive mosquito species in the world [3, 4]. Since the second half of the 20th century, this mosquito species has spread worldwide except for Antarctica [5], probably mainly disseminating through the transportation of eggs *via* the international trade of used tires [3, 6] and exotic plants [7]. Its ecological plasticity in different traits including egg diapause, the ability to use natural or urban larval breeding sites [8, 9] and opportunistic feeding behavior [10, 11] may have aided dispersal and adaptation to newly colonized environments across many contrasting climatic conditions; ranging from tropical to temperate [4]. In addition to its aggressive daytime human-biting behavior, *Ae. albopictus* has been shown in laboratory tests to be a proven competent vector for over 20 arboviruses including yellow fever virus, West Nile virus [8] and Zika virus [12]. The continued activity and dispersal of this mosquito has significant importance to public health, for example, due to its involvement in recent arbovirus outbreaks such as chikungunya outbreaks in Réunion Island in 2005–2006 [13] and in Italy in 2007 [14] and 2017 [15].

Réunion Island, an overseas French territory, is a volcanic island of 2512 km^2^ located about 700 km east of Madagascar. It hosts about 850,000 inhabitants, mainly living in coastal regions. Population density and climate vary greatly across the island: the west coast is dry and densely populated, the east coast is wet with a lower human population density and the center of the island is a humid mountainous region. Twelve mosquito species (from four genera: *Aedes*, *Anopheles*, *Culex* and *Orthopodomyia*) including *Ae. albopictus* and *Ae.* *aegypti* are currently known to inhabit the island [16, 17]. However, *Ae. albopictus* is one of the most abundant mosquito species in Réunion Island and is common all over the island in urban, periurban and rural areas sometimes reaching up 1200 m [17, 18]. This mosquito species was first recorded in Réunion Island in 1913 [19] while the presence of *Ae. aegypti* has been reported since 1902 [20]. *Aedes albopictus* gradually replaced *Ae. aegypti* whose distributional range was considerably reduced [17, 21, 22] probably due to dichlorodiphenyltrichloroethane (DDT) treatments for mosquito-control campaigns against malaria vectors in the 1950s [16].

The Asian tiger mosquito has become a major concern for public health in Réunion Island because it has been recognized as the main vector in several arbovirus outbreaks. In 2005–2006, this island has experienced a chikungunya virus outbreak that was responsible of 265,000 human cases (34% of the population) with 237 associated deaths [23]. More recently, an epidemic caused by a dengue virus type-2 (DENV-2) started at the end of 2017 and is still ongoing on the island [24, 25]. The French public health agency has reported more than 49,000 cases of dengue since the beginning of the epidemic, with most of the cases reported in the south and west coasts of the island [24]. As vector capacity for mosquito-borne viruses depends largely on vector and virus genetics [26–29], we asked whether genetic structure of *Ae. albopictus* populations may explain the observed geographical distribution of human cases of dengue in Réunion Island. Previous studies of *Ae.* *albopictus* population structure from Réunion Island showed contradictory results. Paupy et al. [30] and Delatte et al. [9], using allozymes and microsatellite loci, respectively, found that populations from the west and east coasts were genetically differentiated while, more recently, Sherpa et al. [31] did not find evidence for any genetic structure using 1561 single nucleotide polymorphisms (SNPs).

The present study aims at a better understanding the population structure of *Ae.* *albopictus* in Réunion Island. For this purpose, 564 mosquitoes from 19 populations sampled in different locations across the island (West, East and Center), were genotyped using 16 microsatellite loci that have previously shown their effectiveness in describing population structure of *Ae.* *albopictus* [32–35]. Genetic diversity as well as gene flow among populations were then assessed. This study could help understanding patterns of dengue transmission in Réunion Island in order to implement effective strategies to protect human populations.

## Methods

### Mosquito samples

**Fig. 1:**
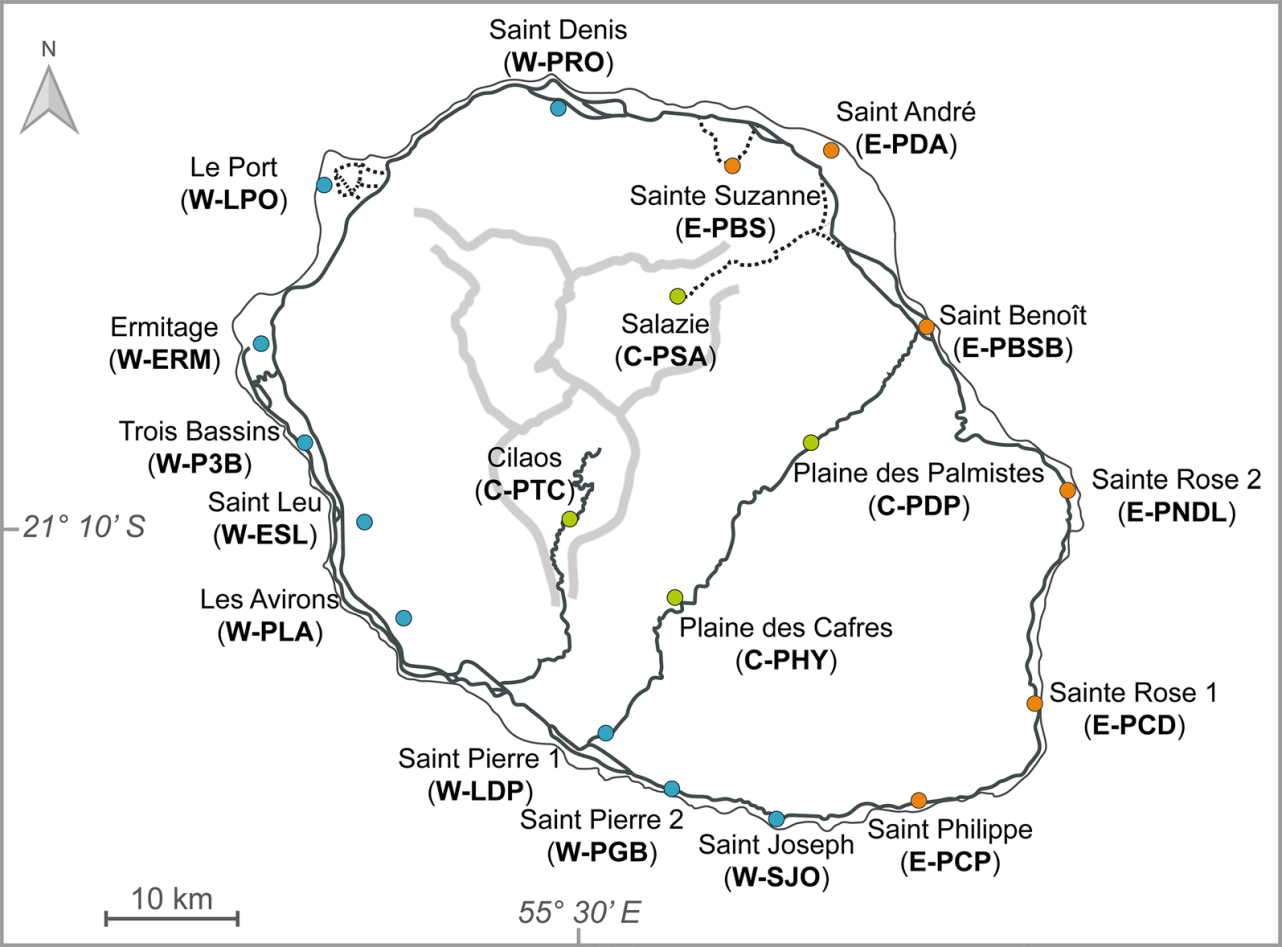
Map of sampling sites where *Aedes albopictus* mosquitoes were collected in Réunion Island. For each site, GPS coordinates and environmental characteristics are indicated in Additional file 1: Table S1. Solid lines: national roads; dotted lines: secondary roads (not all secondary roads are shown); thick grey lines: outline of cirques. The sampling sites are colored according to the climatic regions: blue, West; orange, East; green, Center (see Fig. 2 for more details on climatic regions). Population codes are given in parentheses

*Aedes albopictus* eggs were sampled from 19 sites across Réunion Island (Fig. 1) in November and December 2016 and in February and March 2017. Most of the samples were collected in periurban areas with most buildings being family homes with gardens or allotment gardens. Six ovitraps were randomly placed at each site and spaced 5–10 m apart. Eggs from the six ovitraps (200–1500 eggs) were pooled together according to the site and transported to an insect laboratory where they were reared to adulthood. After morphological identification of adults using Theobald’s criteria [36], individuals were stored at − 20 °C until molecular analyses. Although egg sampling can show some limitation for population genetics due to sampling of siblings, this method was best suited for the collection of the large number of *Ae.* *albopictus* specimens required and at different locations across the island. Each of the 19 sites was characterized according to the following environmental variables: altitude, temperature, rainfall, vegetation type and land cover (Additional file 1: Table S1). We used mean temperatures normalized over 30 years (1981–2010) from the French meteorological weather service (Météo-France, https://donneespubliques.meteofrance.fr/). Rainfall data were provided by climatic stations installed by the French Agricultural Research Centre for International Development (CIRAD). The major type of vegetation around the sampled sites was determined from a mapping established by the Conservatoire Botanique National de Mascarin. Finally, the land-use was assessed using a mapping developed by the CIRAD from satellite data obtained in 2016–2017. Multiple Factor Analysis (MFA) was conducted using the *FactoMineR* package v.1.41 [37] in R software [38] to gather the 19 sites into regions with similar environmental characteristics.

### Microsatellite processing and genotyping

DNA was extracted from individual mosquitoes using a cetyltrimethylammonium bromide (CTAB) protocol [39]. A total of 564 individuals were genotyped (24–30 individuals per site). In each site, half of the analyzed individuals were males and the other half females. A set of 16 microsatellite loci previously developed for *Ae. albopictus* was used [32, 34] (Additional file 2: Table S2). Microsatellite locus primers were pooled in three PCR-mixes based on non-overlapping size ranges and amplified by multiplex PCRs using fluorescent primers (Additional file 2: Table S2). All PCR reactions (10 µl) contained 0.5 U of Taq DNA polymerase, 6 µM of dNTPs, 37.5 µM of MgCl_2_, 1.2–7 µM of each primer (Additional file 2: Table S2) and 10 ng of mosquito DNA. The following program was used to amplify all 16 loci: 10 min at 95 °C followed by 35 cycles at 95 °C for 30 s, 57 °C for 30 s and 72 °C for 1 min and a final elongation step of 72 °C for 10 min. PCR products were run on a 3730XL DNA Genetic Analyzer (Applied Biosystems, California, USA) with a GeneScan 500LIZ size standard. Microsatellite alleles were read using the software GeneMapper v. 4.0 (Applied Biosystems).

### Statistical analyses

#### Genetic diversity

The presence of null alleles was checked for each of the 16 microsatellite loci at the population level using Micro-Checker v.2.2.3 [40]. Multi-locus genotypes (MLGs) were assessed using the *allelematch* R package v.2.5 [38, 41]. The inbreeding coefficient (*F*_*IS*_) was assessed using Arlequin v.3.5 [42]. The average and effective number of alleles (*N*_*a*_ and *N*_*e*_ respectively), the number of private alleles (*N*_*P*_) and the observed and expected heterozygosities (*H*_*O*_ and *H*_*E*_ respectively) were estimated using GenAlEx v.6.5 [43]. Tests of Hardy-Weinberg equilibrium (HWE) and linkage disequilibrium (LD) were performed using *GENEPOP* v.4.2 on the web [44, 45] and *test_LD* functions in the *genepop* R package v.1.05 [44], respectively. Sequential Bonferroni corrections were applied for all multiple comparisons [46].

A large departure from panmixia and strong linkage disequilibrium were detected for all microsatellite loci that might reflect the existence of substructure in the populations. Such a cryptic structure could be due to the sampling method that can favor the sampling of close relatives (i.e. half-sibs or full-sibs). To detect possible close relatives in our samples, individuals of each population were assigned to different genetic clusters, potentially corresponding to families of related individuals, using a hierarchical clustering approach based on principal components analyses (*HCPC* function, the *Facto MineR* R package v.1.41 [37]). To limit the potential bias induced by the sampling of related individuals, 100 subsampled datasets were generated by randomly sampling a maximum of two individuals per genetic cluster in each population. Hardy-Weinberg equilibrium (HWE), linkage disequilibrium (LD), the effective number of alleles (*N*_*e*_) and the expected heterozygosity (*H*_*E*_) were estimated for the 100 subsampled datasets, as described above. Although the subsampling procedure does not solve the problem of sampling relatives, it allows assessment of the potential effect of relatedness in the dataset in population genetic analyses.

#### Population structure

##### Unsupervised clustering

*Aedes* *albopictus* population structure was investigated using principle component analysis and the Bayesian clustering algorithm implemented in STRUCTURE v.2.3.4 [47], with the admixture model considering correlated allele frequencies. The number of groups (K) was varied from 1–19. A ‛burn-in’ period of 10^4^ iterations was followed by 10 runs of 10^5^ iterations leading to the estimation of the membership coefficients. Runs were pooled using the CLUMPAK server [48]. The choice of the best number of clusters (K) was based on the Evanno’s method [49].

##### Population differentiation

For each pair of populations, the fixation index (*F*_*st*_) was computed following Weir & Cokerham method [50]. For each microsatellite locus, the allelic frequencies were computed either (i) without considering individual relatedness using GenAlEx v.6.5 [43] or (ii) by taking the relationships among individuals into account using Colony v.2.0.6.4, likelihood method [51]. *F*_*ST*_ values were computed on the basis of the allelic frequencies estimated with both methods to detect the potential effect of relatedness on the estimation of genetic differentiation.

The distribution of genetic variation among individuals, populations and geographical regions was assessed in hierarchical analyses of molecular variance (AMOVA; [52, 53]) using GenAlEx v.6.5 [43]. Three geographical regions (West, East and Center) were considered according to environmental characterization of the sampling sites.

Isolation by distance was analyzed using R software [38], computing the linear relationship between pairwise estimates of *F*_*ST*_ and the geographical distance by road. Sequential Bonferroni corrections were applied for all multiple comparisons [46].

## Results

**Fig. 2:**
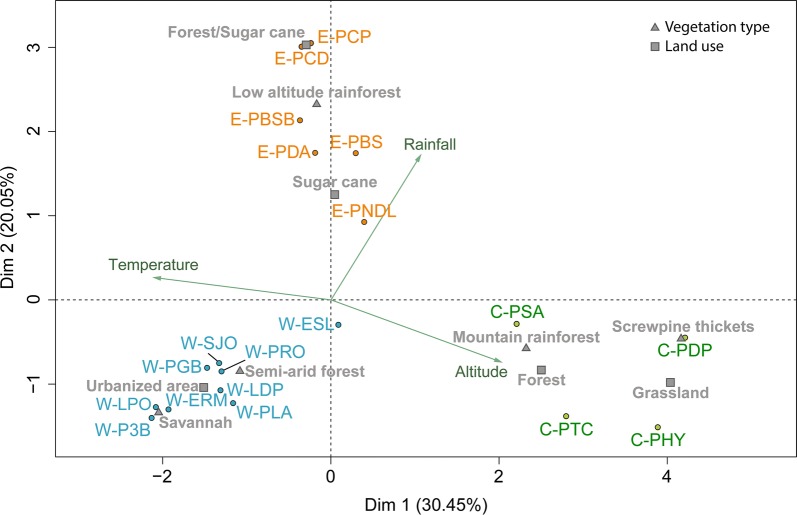
A two-dimensional plot from a multiple factor analysis (MFA) performed on the 19 *Aedes albopictus* sampling sites. The analysis was based on five environmental variables: two climatic variables (temperature and rainfall), altitude, land use and type of vegetation

To characterize the genetic differentiation of *Ae.* *albopictus* populations, mosquitoes from 19 sites across Réunion Island were genotyped (Fig. 1). To cluster the sites according to their environmental characteristics, multiple factor analysis (MFA) was performed based on five factors: two climatic variables (temperature and rainfall), altitude, land use and the type of vegetation (Fig. 2). The five factors contributed equivalently to the first axis of the MFA. The land-use and the vegetation type contributed more to the second axis (37% for each of the two factors) than the climatic variables (21% for both temperature and rainfall) and the altitude (5%). The first axis of the MFA divided the sites according to the temperature and the altitude, while the second axis divided the sites according to the rainfall. The 19 sites were thus clustered into three regions (Fig. 2): the West coast region with nine sites (W-PRO, W-LPO, W-ERM, W-P3B, W-ESL, W-PLA, W-LDP, W-PGB and W-SJO); the East coast region with six sites (E-PDA, E-PBS, E-PBSB, E-PNDL, E-PCD and E-PCP) and the Center region with four sites (C-PTC, C-PHY, C-PDP and C-PSA). The west coast was characterized by altitude ranging from 9–437 m a.s.l., temperatures from 19–25 °C and rainfall from 490–1540 mm (Additional file 1: Table S1). The east coast was wetter than the west coast and the center, with altitude ranging from 20–314 m a.s.l., temperatures from 20–24 °C and rainfall from 2130–3700 mm. The center of the island was mainly characterized by sites with high altitude, ranging from 449–1161 m a.s.l., temperatures from 14–19 °C and rainfall from 1150–3830 mm.

### High genetic diversity of *Ae. albopictus* in Réunion Island

Overall 564 mosquitoes from the 19 sites (24–30 mosquitoes per site) were genotyped using 16 microsatellite loci [32, 34]. For six microsatellite loci (Aealbmic4, Aealbmic5, Aealbmic12, Aealbmic13, Albdi6, Albtri3), null alleles were observed in more than 70% of the populations (Additional file 3: Table S3). These six microsatellite loci were therefore removed from downstream population genetics analyses. We also removed 32 individuals with two or more missing alleles in their multilocus genotypes (MLGs). Among the remaining 532 individuals, 530 different MLGs (i.e. differentiated by at least one allele) were identified; 528 were unique (i.e. identified in only one individual), while two MLGs were shared, each by two mosquitoes from the same population. Only unique MLGs were kept for population genetics analyses. Thus, our final dataset consisted of 530 individuals genotyped with 10 microsatellite loci (Aealbmic2, Aealbmic3, Aealbmic6, Aealbmic7, Aealbmic8, Aealbmic9, Aealbmic10, Aealbmic11, Aealbmic16 and Albtri45).

Allelic richness of the 10 microsatellite loci was high with the number of alleles per locus varying from 2 (Aealbmic2) to 15 (Aealbmic3) and the average number of alleles per population (*N*_*a*_) varying from 4.70 (in W-P3B) to 5.90 (in W-LDP and W-PRO; Additional file 4: Table S4).

### Cryptic intrapopulation structure due to individual relatedness

**Fig. 3:**
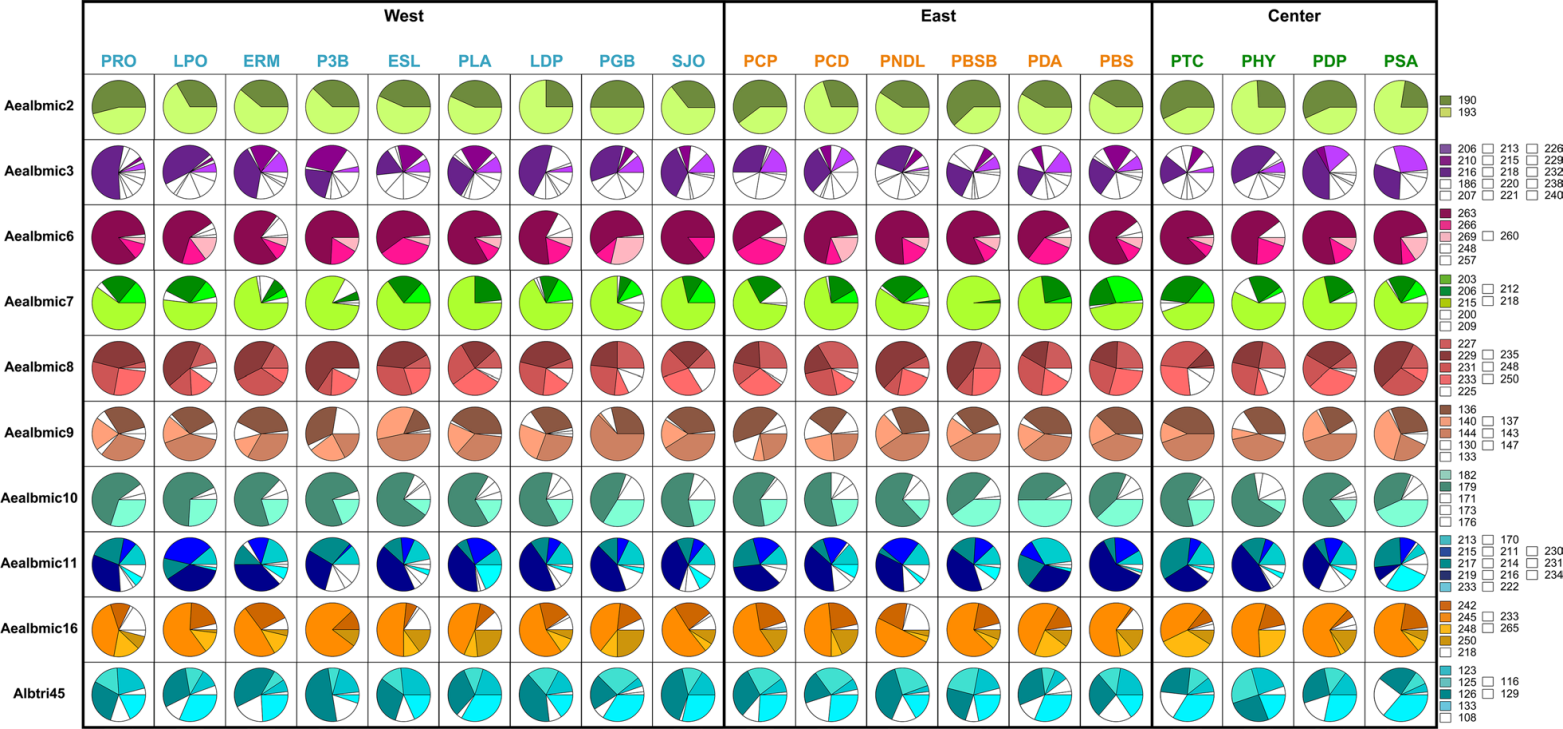
Allelic frequencies for 10 microsatellite loci in the 19 mosquito populations. Only alleles with a frequency of at least 25% in one or more populations are colored. Numbers correspond to the size of alleles for each microsatellite locus

In each population, the average number of effective alleles (*N*_*e*_) was lower than the observed average number of alleles (*N*_*a*_) thus reflecting imbalanced allelic frequencies in populations. Indeed, for most of the loci, one to three alleles were overrepresented within populations (Fig. 3). Besides, almost all alleles were shared by mosquitoes from different populations, very few private alleles were observed: two in W-ERM (Aealbmic11 and Aealbmic3) and in W-PRO (Aealbmic16 and Aealbmic9) and one in W-LPO, C-PHY (Aealbmic8) and in C-PSA (Aealbmic11; Additional file 4: Table S4). The observed heterozygosity (*H*_*O*_) varied from 0.47–0.62 while the expected heterozygosity (*H*_*E*_) varied from 0.55–0.66. In all populations, *H*_*E*_ was higher than *H*_*O*_ and the inbreeding coefficients (*F*_*IS*_) were significantly positive, indicating a deficit in heterozygotes except for E-PNDL and C-PHY for which it was not significantly different from zero (Additional file 4: Table S4). Moreover, significant linkage disequilibrium was observed between all pairs of loci (Chi-square test; 46 < *χ*^2^ < 127; *df* = 38; *P* < 0.013). However, no deviation from HWE and no evidence of linkage disequilibrium were observed after sequential Bonferroni correction for multiple testing.

We therefore examined whether there were clusters of individuals within each population. For this purpose, we used a hierarchical clustering approach based on principal components analyses (HCPC) that grouped individuals according to their genetic proximity (Additional file 5: Figure S1). Two (E-PBS, E-PCP and C-PDP) to six (E-PBSB) clusters (with 1–24 individuals per cluster) were found per population (Table 1). The presence of clusters of individuals within each population suggests the presence of relatives. A high number of relatives within each genetic cluster may explain the observed deviations from HWE and the significant linkage disequilibrium between microsatellite markers. To break down this clustering effect due to individualsʼ relatedness (e.g. half-sib structure), we generated subsampled datasets by randomly sampling only two individuals per genetic cluster within each population (except for clusters with only one individual). Overall 100 subsampled datasets (with 4–10 individuals per population and 118 individuals per dataset) were generated. Each subsampled dataset was further used to perform population genetics analyses. Low sample size per population was used (4–10 individuals, i.e. no more than 2 individuals per cluster) because our preliminary analyses showed that including a higher number of individuals from the same cluster increased the probability to observe a departure from panmixia. Most of the populations appeared to be at HWE (Table 1). Indeed, all populations were at HWE in more than 60 subsampled datasets over the 100 subsampled datasets tested for HWE tests, except W-LDP. In addition, no linkage disequilibrium was observed between all pairs of loci (Additional file 6: Figure S2).

**Table 1:** Summary statistics in each *Aedes albopictus* population estimated using the 100 subsampled datasets

Region	Population	*n*	*N*_*c*_	*N*_*i*_	*N*_*e*_ ± *SE*	*H*_*O*_	*H*_*E*_ ± *SE*	*n*_*HWE*_
West	PRO	25	3	6	2.83 ± 0.35	0.60 ± 0.06	0.59 ± 0.05	100
	LPO	27	4	8	2.94 ± 0.34	0.58 ± 0.06	0.61 ± 0.05	100
	ERM	27	3	6	2.79 ± 0.32	0.60 ± 0.04	0.59 ± 0.05	96
	P3B	29	4	8	2.69 ± 0.44	0.46 ± 0.04	0.54 ± 0.06	64
	ESL	30	4	8	3.05 ± 0.44	0.60 ± 0.04	0.62 ± 0.05	88
	PLA	30	4	7	2.93 ± 0.37	0.56 ± 0.05	0.61 ± 0.05	100
	LDP	26	3	6	2.84 ± 0.31	0.58 ± 0.05	0.60 ± 0.05	44
	PGB	28	3	6	3.01 ± 0.36	0.60 ± 0.06	0.62 ± 0.04	88
	SJO	28	3	6	2.85 ± 0.36	0.61 ± 0.04	0.60 ± 0.05	100
East	PCP	29	2	4	2.73 ± 0.30	0.63 ± 0.08	0.58 ± 0.05	100
	PCD	30	3	6	3.20 ± 0.44	0.59 ± 0.06	0.62 ± 0.06	100
	PNDL	27	3	6	2.98 ± 0.40	0.63 ± 0.04	0.61 ± 0.04	92
	PBSB	25	6	10	2.97 ± 0.46	0.47 ± 0.03	0.57 ± 0.07	100
	PDA	24	3	6	3.00 ± 0.41	0.60 ± 0.04	0.61 ± 0.05	88
	PBS	29	2	4	2.61 ± 0.32	0.52 ± 0.08	0.56 ± 0.05	100
Center	PTC	28	3	5	2.86 ± 0.37	0.61 ± 0.04	0.59 ± 0.05	100
	PHY	29	3	6	2.81 ± 0.31	0.62 ± 0.06	0.60 ± 0.05	96
	PDP	30	2	4	2.50 ± 0.33	0.60 ± 0.05	0.52 ± 0.07	100
	PSA	29	3	6	2.94 ± 0.30	0.66 ± 0.04	0.62 ± 0.04	100

*Abbreviations*: *n*, number of individuals analyzed per population; *N*_*C*_, number of clusters generated by HCPC analyses; *N*_*i*_, number of individuals kept in each population (corresponding to two individuals per cluster, except for clusters with only one individual) for each of the 100 subsampled datasets; *N*_*e*_, mean expected number of alleles; *H*_*O*_ and *H*_*E*_, mean observed and expected heterozygosities over all loci, respectively; *n*_*HWE*_, number of subsampled datasets (over the 100 generated) for which Hardy-Weinberg equilibrium test was found non-significant (i.e. population at the equilibrium); SE, standard error

### No genetic structure of *Ae. albopictus* populations

The fixation index, *F*_*ST*_, was then used to investigate population differentiation. For each pair of populations, *F*_*ST*_ was computed by using the full dataset directly or the likelihood method (see “Methods” section) that allows relatedness among individuals to be taken into account when estimating the allelic frequencies. Both methods showed similar results (Pearson’s product moment correlation, *R*^2^ = 0.79; *P* < 0.001; *n* = 171; Additional file 7: Figure S3). Therefore, only the results obtained with the full dataset (i.e. without taking relatedness among individuals into account) were considered. The *F*_*ST*_ values ranged from 0.011–0.057 and were significant in most pairwise comparisons (Table 2). However, no pairwise comparison remained significantly different from 0 after the sequential Bonferroni correction. The average *F*_*ST*_ among populations of the West coast region (*F*_*ST*_ = 0.02 ± 0.01) was lower than that measured among populations of the East coast or Center (*F*_*ST*_ = 0.03 ± 0.004 and 0.04 ± 0.01, respectively). A linear regression model between matrices of *F*_*ST*_ and geographical distances was not significant (*R*^2^ = 1.36 × 10^−4^, *P* = 0.88; *n* = 171; Additional file 8: Figure S4).

**Table 2:** Pairwise *F*_*ST*_ between the 19 *Aedes albopictus* populations calculated using the Weir and Cockerham’s method

Regions	Populations	West									East						Center			
		PRO	LPO	ERM	P3B	ESL	PLA	LDP	PGB	SJO	PCP	PCD	PNDL	PBSB	PDA	PBS	PTC	PHY	PDP	PSA
West	PRO		*	ns	***	**	*	ns	***	ns	***	**	*	**	**	**	**	***	ns	***
	LPO	0.018		ns	***	**	**	ns	***	*	***	*	ns	***	**	**	***	*	**	***
	ERM	0.016	0.015		***	**	*	ns	**	ns	***	*	*	**	*	**	***	*	***	***
	P3B	0.035	0.036	0.027		***	***	***	***	***	***	***	***	***	***	***	***	***	***	***
	ESL	0.021	0.022	0.021	0.032		**	*	***	**	***	***	**	***	***	**	***	***	**	***
	PLA	0.017	0.021	0.016	0.037	0.018		***	**	ns	**	**	**	***	**	**	***	***	*	***
	LDP	0.017	0.015	0.012	0.029	0.018	0.021		***	ns	***	*	*	***	**	**	***	ns	**	**
	PGB	0.024	0.024	0.024	0.057	0.029	0.024	0.028		***	***	*	***	**	*	***	**	***	***	***
	SJO	0.015	0.017	0.014	0.033	0.019	0.011	0.015	0.023		***	*	*	***	*	**	***	**	**	***
East	PCP	0.026	0.025	0.025	0.044	0.028	0.023	0.029	0.025	0.026		**	***	***	**	***	***	***	***	***
	PCD	0.023	0.018	0.016	0.040	0.024	0.017	0.016	0.018	0.016	0.023		**	***	*	**	***	**	***	**
	PNDL	0.017	0.014	0.017	0.030	0.018	0.020	0.017	0.029	0.017	0.026	0.021		**	**	***	***	**	**	***
	PBSB	0.023	0.033	0.028	0.037	0.032	0.024	0.032	0.026	0.023	0.028	0.029	0.030		**	***	***	***	**	***
	PDA	0.024	0.023	0.020	0.046	0.025	0.022	0.024	0.020	0.020	0.021	0.019	0.032	0.025		*	**	***	***	**
	PBS	0.022	0.022	0.025	0.047	0.022	0.019	0.024	0.025	0.020	0.030	0.022	0.027	0.031	0.021		***	***	***	***
Center	PTC	0.021	0.031	0.029	0.057	0.031	0.023	0.037	0.030	0.026	0.034	0.032	0.032	0.043	0.025	0.028		***	***	***
	PHY	0.024	0.017	0.017	0.045	0.023	0.024	0.013	0.025	0.018	0.027	0.019	0.023	0.040	0.026	0.026	0.032		***	***
	PDP	0.015	0.020	0.021	0.033	0.018	0.014	0.024	0.025	0.017	0.022	0.022	0.018	0.021	0.029	0.027	0.028	0.028		***
	PSA	0.033	0.028	0.026	0.045	0.040	0.036	0.029	0.042	0.029	0.045	0.023	0.036	0.043	0.025	0.037	0.045	0.038	0.039	

*Notes*: Below the diagonal: *F*_*ST*_ values; above the diagonal: *F*_*ST*_ significance. ns: non-significant *F*_*ST*_ values (permutation test, *P* ≤ 0.05). No pairwise *F*_*ST*_ values remained significantly different from 0 when using sequential Bonferroni correction for multiple comparisons

*P < 0.05, **P < 0.001, ***P < 0.001

**Fig. 4:**
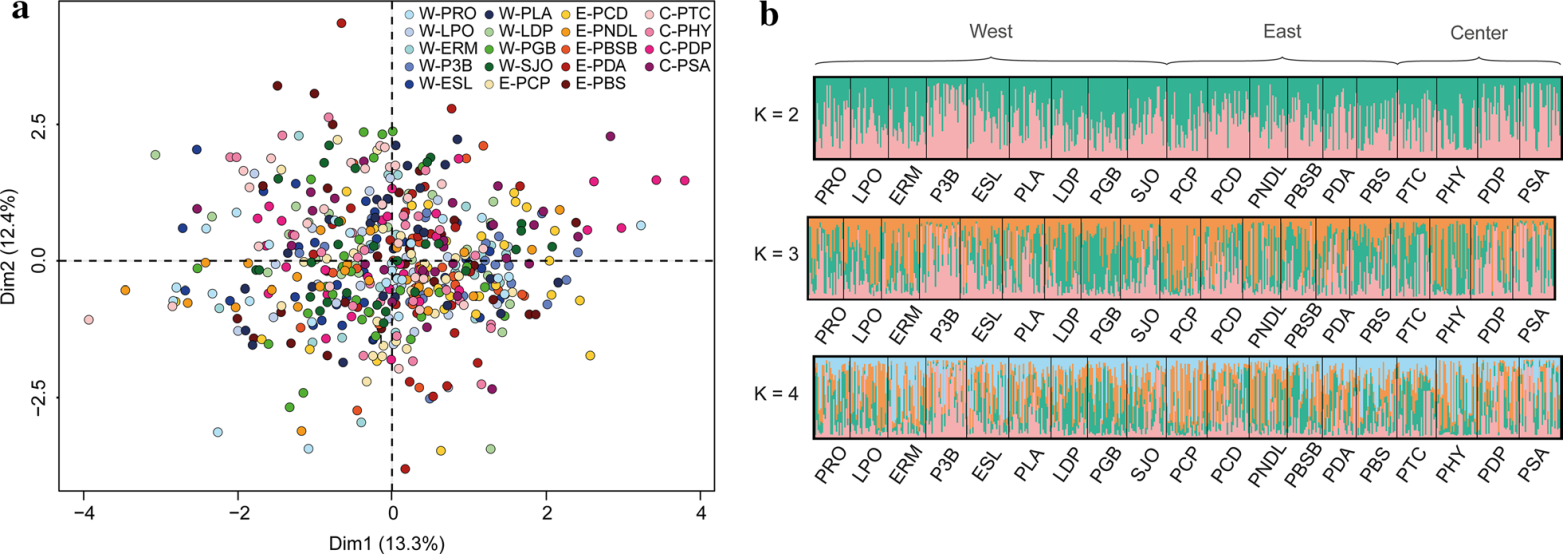
Genetic structure of *Aedes albopictus* in Réunion Island. **a** A two-dimensional plot from a principal component analysis (PCA) based on individual genotypes for 10 microsatellite loci. **b** Bayesian analysis of population structure for the 19 mosquito populations produced using STRUCTURE. Each vertical bar represents an individual, each color represents a cluster (K) and the color of the bar indicates the probability of assignment to each cluster. Only the results for K = 2 to K = 4 are presented

Analysis of molecular variance (AMOVA; Table 3) across the 19 *Ae. albopictus* populations considering the three regions (West, East and Center) revealed that 97% of the variance was found among individuals within populations (*P* = 0.001), while only 3% was found between populations (*P* = 0.001) and 0% between regions (*P* = 0.80; Table 3). Finally, neither principal components analysis (Fig. 4a) nor Bayesian clustering approach (STRUCTURE v.2.3.4; Fig. 4b) revealed any population structure.

**Table 3:** Analyses of molecular variance (AMOVA) for the 19 *Aedes albopictus* populations

Source	*df*	SS	Variation (%)	*P*-value
Among regions	2	17.83	0	0.796
Among populations within regions	16	150.97	3	0.001
Among individuals within populations	1041	3336.61	97	0.001
Total	1059	3505.41	100	

*Abbreviations*: *df*, degrees of freedom; SS, sum of squares

## Discussion

We assessed the genetic diversity and the genetic structure of 19 *Ae.* *albopictus* populations from Réunion Island using 10 microsatellite loci. We highlighted a high genetic diversity but found no evidence of genetic differentiation between populations despite the contrasted environments between the three regions (West, East and Center) where mosquitoes were collected.

### A high genetic diversity reflecting long-term established mosquito populations

The genetic diversity observed in the 19 *Ae. albopictus* populations was high: on average more than five alleles were observed per population and 530 MLGs were identified among the 532 mosquitoes examined. Using microsatellite loci, Manni et al. [35] observed a higher genetic diversity in *Ae. albopictus* populations from Réunion Island compared to populations from Asia (e.g. from Japan and China), Europe (from Greece, Albania, Italy) and America (from Virginia, USA). A recent study based on 1561 genome-wide SNPs also described a higher genetic diversity in *Ae. albopictus* populations from Réunion Island compared to European populations [31]. Two main hypotheses may explain the highest genetic diversity of *Ae. albopictus* populations from Réunion Island. The first hypothesis is that *Ae. albopictus* populations in Réunion Island result from an admixture of several populations from different geographical origins. According to Manni et al. [35] investigations of population genetics and Approximate Bayesian Computation (ABC) analyses, *Ae. albopictus* populations from Réunion Island might result from admixture between populations from Thailand and Japan. By comparing mtDNA polymorphism between ancient (collected in 1956) and more recent samples (collected in 2007), Delatte et al. [54] suggested that two waves of *Ae. albopictus* invasion may have occurred in Réunion Island, the first one several centuries ago and the second one in the 1990s, leading to a replacement of populations. The assumption of population replacement from 1990s seems an unlikely explanation for our results because the genetic diversity in Réunion Island is higher than observed in Albania [31, 35] where *Ae. albopictus* has been recorded since 1979 [55] or in Italy where this mosquito species has been established since 1990 [56]. However, it is likely that several introduction events of *Ae. albopictus* individuals from different geographical origins might have occurred in Réunion Island. The second hypothesis is that long-term established mosquito populations would have favored the accumulation of genetic variability within populations, countering the effects of any initial demographic bottleneck. This would be supported by the fact that *Ae. albopictus* was first recorded in Réunion Island in 1913 [19] and it became one of the most abundant species in the island.

### No genetic structure revealing the impact of humans in the distribution of *Ae. albopictus*

The analysis of the 19 mosquito populations with the 10 genotyped microsatellite loci revealed significant linkage disequilibrium between all pairs of loci. This could be explained by the imbalanced allelic frequencies observed in all populations and for all loci: the most frequent alleles are more often associated, regardless of their position on chromosomes. The over-representation of some alleles may be due to a founder effect (only few individuals have contributed to the establishment of the populations) but may also reflect the fact that each of the sampled populations was probably represented by the progenies from few females. Indeed, we measured significant positive *F*_*IS*_ in almost all populations reflecting a deficit in heterozygotes. Ignoring closely related individuals can lead to bias in the estimation of allelic frequencies and thus in the resulting population genetics analyses such as tests of HWE and linkage disequilibrium [57]. In this study, the use of the full dataset for population genetics analyses led to significant deviations from HWE and linkage disequilibrium. However, when genetically close related individuals were removed from the dataset, almost all populations were in panmixia and no linkage disequilibrium was observed between pairs of microsatellite loci. Previous studies based on allozymes also reported heterozygote deficits leading to significant deviation from HWE [30, 58–62]. Significant positive *F*_*IS*_ values were also observed in several studies based on microsatellites (*F*_*IS*_ ranging from 0.10–0.20; [9, 33, 34]) and SNPs (mean *F*_*IS*_ of 0.19 for populations from Réunion Island; [31]) in populations from both native and non-native areas of the Asian tiger mosquito. These positive *F*_*IS*_ values could be explained by inbreeding resulting from preferential mating among relatives or by sampling methods [63]. Indeed, we used samples collected as eggs in ovitraps (six ovitraps for each site) and eggs collected in the same ovitrap could belong to the same progeny. Moreover, *Ae. albopictus* has low dispersal capacities (lower than 500 m) [64, 65] and females can lay eggs in several containers [66]. Hence, it is likely that very few females laid eggs in the six ovitraps used in each sampled site. Therefore, although we genotyped a high number of mosquitoes in each population (24–30 mosquitoes per population), it is likely that this does not represent the sampling site diversity. In future investigations, it would be interesting to use adults (which is not straightforward with *Ae. albopictus*) or eggs sampled at different times to increase the number of sampled progenies. Anyway, we recommend testing for relatedness between individuals when significantly positive *F*_*IS*_ are detected in populations. This is all the more relevant given the rapid spread of *Ae. albopictus* and its involvement in arbovirus outbreaks that leads to an increasing number of studies to understand its genetics and adaptation.

Both the Bayesian clustering algorithm implemented in STRUCTURE and the analysis of molecular variance (AMOVA) revealed an absence of population structure despite a high genetic diversity and a great variability of bioclimatic conditions (climate, vegetation type or anthropogenic activities). This suggests frequent exchange between individuals from different locations across the island. The absence of isolation by distance strongly suggested that human-assisted long-distance gene flows are frequent and play a key role in the dispersal of the Asian tiger mosquito in Réunion Island as has already been shown elsewhere at both large [67, 68] and local scales [69]. Our results are consistent with those of Sherpa et al. [31] who did not detect differentiation among populations from Réunion Island using thousands of genome-wide SNPs. However, previous investigations in Réunion Island using allozymes [30] or microsatellite loci (designed for *Ae. aegypti* [9]) highlighted genetic differentiation between *Ae. albopictus* populations from the east and west coasts related to ecological factors [9, 30]. Apart from molecular markers, the difference between these previous studies and our results could be explained by sampling methods. Indeed, we used only eggs from ovitraps (which are artificial sites) that were laid in periurban areas whereas different stages (larvae, pupae and adults) collected in both natural (gully, rock or tree holes) and artificial (tires or flower pots) sites were used in these previous investigations [9, 30].

## Conclusions

In this study, we observed a high genetic diversity in *Ae. albopictus* populations from Réunion Island, probably due to their long-term establishment. We also observed a lack of genetic structure reflecting a strong connectivity among populations. The absence of population structure and isolation by distance represent key information for the management of vector control strategies against *Ae. albopictus* in Réunion Island. Vector control strategies could be applied at the island scale by taking into account environmental characteristics and mosquito population densities through time because gene flow between mosquito populations may have a limited impact in their effectiveness. Additionally, these results increase our understanding of the evolutionary history of the Asian tiger mosquito in Réunion Island and will allow deciphering its vector capacity. Further studies on vector competence using the epidemic DENV-2 strain and *Ae. albopictus* populations sampled across Réunion Island should help understanding the transmission patterns of dengue virus currently causing an outbreak in the island. As vector capacity can also be affected by extrinsic or abiotic factors such as temperature, rainfall, vector density, vector survival and the probability a vector feeds on a host [29], these factors should also be taken into account to better understand the transmission patterns of dengue virus in Réunion Island.

## Supplementary information

**Additional file 1: Table S1**. Environmental characteristics of the 19 sampling sites of *Aedes albopictus* in Réunion Island.

**Additional file 2: Table S2**. Information on the microsatellite loci used for the genotyping of *Aedes albopictus* populations. *Abbreviations*: F, forward; R, reverse.

**Additional file 3: Table S3**. Presence/absence of null alleles for each microsatellite locus in the 19 mosquito populations.

**Additional file 4: Table S4**. Microsatellite variation in the 19 *Aedes albopictus* populations. *Abbreviations*: *n*, number of individuals analyzed; *N*_*a*_, number of alleles; *N*_*e*_, effective number of alleles; *N*_*p*_, number of private alleles; *H*_*O*_, observed heterozygosities; *H*_*E*_, expected heterozygosities; *F*_*IS*_, inbreeding coefficient (*P** < **0.05, **P** < **0.01, ***P** < **0.001); SE, standard error.

**Additional file 5: Figure S1**. Hierarchical clustering of *Aedes albopictus* individuals from the same population. Example showing the case of the population C-PSA (*n*** = **25 individuals). a Dendrogram generated by the hierarchical clustering. b Principal component analysis. *Abbreviation*: Ind, individual.

**Additional file 6: Figure S2**. Distribution of linkage disequilibrium Chi-square statistic estimated for each couple of loci. In grey: over the 100 subsampled datasets; in black: for the full dataset.

**Additional file 7: Figure S3.** Relationship between *F*_*ST*_ values for *Aedes albopictus* pairs of populations. a Comparisons of *F*_*ST*_ estimated using R (x-axis) and GenAlEx v.6.5 [43] (y-axis) without taking into account relatives. b Comparisons of *F*_*ST*_ estimated using R without taking relatives into account (x-axis) and by taking relatives into account (y-axis). Full line: regression line; dotted line: y** = **x.

**Additional file 8: Figure S4.** Relationship between the *F*_*ST*_ and the distances by road calculated between pairs of mosquito populations. *y*** = **− 3.58 × 10^−6^ *x* + 2.61 × 10^−2^ (Pearsonʼs test: *n*** = **171, *t*** = **− 0.15, *R*^2^** = **1.36 × 10^−4^, *P*** = **0.88).

## Data Availability

Data supporting the conclusions of this article are included within the article and its additional files. Raw data are available on Zenodo (10.5281/zenodo.3475984).

## Abbreviations

HCPC: hierarchical clustering approach based on principal components analyses
DENV–2: dengue virus type-2
SNPs: single nucleotide polymorphisms
MFA: multiple factor analysis
MLGs: multilocus genotypes
HWE: Hardy-Weinberg equilibrium
LD: linkage disequilibrium
