# Supplementary material for: High genetic diversity but no geographical structure of Aedes albopictus populations in Réunion Island
Source: Parasit Vectors. 2019 Dec 19;12:597. doi: 10.1186/s13071-019-3840-x (PMC6924041; doi:10.1186/s13071-019-3840-x)
Supplement: Supplementary file 1 — Additional file 1: Table S1. Environmental characteristics of the 19 sampling sites of Aedes albopictus in Réunion Island. [file 13071_2019_3840_MOESM1_ESM.doc]

**Additional file 1: Table S1**. Environmental characteristics of the 19 sampling sites of *Aedes albopictus* in Reéunion Island.

| Region | Site | X | Y | Altitude (m) | Mean temperature (°C) | Mean rainfall (mm) | Vegetation type | Land use |
| --- | --- | --- | --- | --- | --- | --- | --- | --- |
| West | PRO | 55.4564 | -20.8965 | 36 | 24.25 | 1340 | Semi-arid forest | Urbanized area |
|  | LPO | 55.2875 | -20.9493 | 9 | 25.45 | 490 | Savannah | Urbanized area |
|  | ERM | 55.2326 | -21.0535 | 67 | 24.95 | 540 | Savannah | Urbanized area |
|  | P3B | 55.2647 | -21.125 | 35 | 24.95 | 640 | Semi-arid forest | Savannah |
|  | ESL | 55.3091 | -21.1739 | 437 | 19.1 | 970 | Semi-arid forest | Sugar cane |
|  | PLA | 55.3366 | -21.243 | 331 | 24 | 660 | Savannah | Urbanized area |
|  | LDP | 55.4859 | -21.3231 | 136 | 23.3 | 800 | Semi-arid forest | Urbanized area |
|  | PGB | 55.5332 | -21.3615 | 75 | 23.3 | 1340 | Savannah | Urbanized area |
|  | SJO | 55.6114 | -21.3862 | 28 | 23.3 | 1540 | Low altitude rainforest | Urbanized area |
| East | PCP | 55.7125 | -21.3732 | 46 | 23.15 | 3460 | Low altitude rainforest | Forest/Sugar cane |
|  | PCD | 55.8011 | -21.3057 | 124 | 23.45 | 3700 | Low altitude rainforest | Forest/Sugar cane |
|  | PNDL | 55.8243 | -21.1653 | 60 | 23.45 | 3250 | Low altitude rainforest | Sugar cane |
|  | PBSB | 55.7232 | -21.0488 | 43 | 23.55 | 3110 | Low altitude rainforest | Sugar cane |
|  | PDA | 55.6565 | -20.9262 | 20 | 20.5 | 2130 | Low altitude rainforest | Sugar cane |
|  | PBS | 55.5827 | -20.9324 | 314 | 20.5 | 2630 | Low altitude rainforest | Sugar cane |
| Center | PTC | 55.461 | -21.1772 | 972 | 16.7 | 1150 | Mountain rainforest | Forest |
|  | PHY | 55.5384 | -21.2219 | 1161 | 13.9 | 1440 | Mountain rainforest | Grassland |
|  | PDP | 55.6407 | -21.1276 | 948 | 16.75 | 3830 | Screwpine thickets | Grassland |
|  | PSA | 55.5441 | -21.0242 | 449 | 18.75 | 3280 | Mountain rainforest | Forest |
