# Supplementary material for: High genetic diversity but no geographical structure of Aedes albopictus populations in Réunion Island
Source: Parasit Vectors. 2019 Dec 19;12:597. doi: 10.1186/s13071-019-3840-x (PMC6924041; doi:10.1186/s13071-019-3840-x)
Supplement: Supplementary file 2 — Additional file 2: Table S2. Information on the microsatellite loci used for the genotyping of Aedes albopictus populations. Abbreviations: F, forward; R, reverse. [file 13071_2019_3840_MOESM2_ESM.doc]

**Additional file 2: Table S2.** Information on microsatellite loci used for the genotyping of *Aedes albopictus* populations. *Abbreviations*: F, forward; R, reverse.

| **Marker** | **Sequence** | **Size range (bp)** | **Dye** | **Multiplex** | **Quantity  (**µM**)** | **References** |
| --- | --- | --- | --- | --- | --- | --- |
| Aealbmic3 | F: ACCATACAGCCTGGAGTTCG R: GGGGTTGTGTGAATTGTCGT | 206-238 | VIC | M1 | 2 | [1] |
| Aealbmic6 | F: GATGGTCCGTATTTGGGTTG  R: ATCTTCACTCATCCGCCATC | 248-269 | PET | M1 | 4 | [1] |
| Aealbmic8 | F: TTGTTGTTCGGTTGTTGTTTG  R: CGGGTTCCAACTATGTACGA | 225-235 | 6FAM | M1 | 6 | [1] |
| Albtri3 | F: AGATGTGTCGCAATGCTTCC  R: GATTCGGTGATGTTGAGGCC | 119-131 | PET | M1 | 2 | [2] |
| Albtri45 | F: TTTCAGCTCGGTGTTATGGC  R: TGATGTTGATGATGATGACTACGA | 116-133 | 6FAM | M1 | 2 | [2] |
| Aealbmic4 | F: ATCGCGGGTTTTCTATTCCT  R: ATCAACGAAACCGAAAGCAT | 172-187 | NED | M2 | 1.6 | [1] |
| Aealbmic5 | F: AACCCATCGAACACAGAAGG  R: GTACGGTTGACTCGCTGTGA | 148-222 | 6FAM | M2 | 1.6 | [1] |
| Aealbmic7 | F: ATAGACGGGAGTCGGTTCCT  R: TCCAACCGCTAGTGTCATCA | 200-215 | PET | M2 | 1.6 | [1] |
| Aealbmic13 | F: TCACACCATGGTCAAAGCAT  R: TGCTGAGTTGAATGGAAACG | 142-155 | VIC | M2 | 2.5 | [1] |
| Albdi6 | F: TCTTCATCTACGCTGTGCTC  R: GACGCCAATCCGACAAAGTC | 259-276 | VIC | M2 | 2.5 | [2] |
| Aealbmic2 | F: ACGATGCGTAACCATTCGAT  R: AACACCGCCGAATATGAAAC | 190-193 | PET | M3 | 2.5 | [1] |
| Aealbmic9 | F: GCGATGACAGTGGAACAAGA  R: GCTTGGCAGGGAACAAATTA | 133-147 | VIC | M3 | 1.2 | [1] |
| Aealbmic10 | F: ATCGCCTTCACTCTTCTTCG  R: CCAATCCTGAGCCGTACATT | 171-182 | 6FAM | M3 | 4 | [1] |
| Aealbmic11 | F: CTCTGCGTTCCGGTTCTATC  R: AGGCAACCTCTCGAATGAAA | 213-234 | VIC | M3 | 7 | [1] |
| Aealbmic12 | F: AGAGCCCTCGAAAAGAGAGC  R: AGCACTCATTCTTGGCTTGG | 158-184 | NED | M3 | 3 | [1] |
| Aealbmic16 | F: CACAACAACGAGAGTGTCGAA  R: CCGAGGGCAACACGATATAC | 218-250 | 6FAM | M3 | 5.6 | [1] |

**References**

1. Manni M, Gomulski LM, Aketarawong N, Tait G, Scolari F, Somboon P, et al. Molecular markers for analyses of intraspecific genetic diversity in the asian tiger mosquito, *Aedes albopictus*. Parasit Vectors. 2015;8:1–11.

2. Beebe NW, Ambrose L, Hill LA, Davis JB, Hapgood G, Cooper RD, et al. Tracing the tiger : Population genetics provides valuable insights into the *Aedes* (Stegomyia) *albopictus* invasion of the australasian region. PLoS Negl Trop Dis. 2013;7:e2361.

3. Peakall R, Smouse PE. GenAlEx 6.5: genetic analysis in Excel. Population genetic software for teaching and research-an update. Bioinformatics. 2012;28:2537–9.
