# Supplementary material for: High genetic diversity but no geographical structure of Aedes albopictus populations in Réunion Island
Source: Parasit Vectors. 2019 Dec 19;12:597. doi: 10.1186/s13071-019-3840-x (PMC6924041; doi:10.1186/s13071-019-3840-x)
Supplement: Supplementary file 3 — Additional file 3: Table S3. Presence/absence of null alleles for each microsatellite locus in the 19 mosquito populations. [file 13071_2019_3840_MOESM3_ESM.doc]

**Additional file 3: Table S3.** Presence/absence of null alleles for each microsatellite locus in the 19 mosquito populations.

|  |  | Aealbmic | | | | | | | | | | | | | | Alb | | |
| --- | --- | --- | --- | --- | --- | --- | --- | --- | --- | --- | --- | --- | --- | --- | --- | --- | --- | --- |
| Region | Population | 2 | 3 | 4 | 5 | 6 | 7 | 8 | 9 | 10 | 11 | 12 | 13 | 16 | di6 | | tri3 | tri45 |
| West | PRO |  |  | ● | ● |  |  |  |  |  |  | ● | ● |  | ● | | ● |  |
|  | LPO |  |  | ● |  |  |  | ● |  |  | ● | ● | ● |  | ● | | ● |  |
|  | ERM |  |  | ● |  |  |  |  |  | ● |  | ● |  |  | ● | | ● |  |
|  | P3B |  |  | ● | ● |  |  | ● | ● | ● | ● | ● |  |  | ● | | ● |  |
|  | ESL |  |  | ● | ● |  |  | ● |  | ● | ● | ● | ● |  | ● | | ● |  |
|  | PLA |  |  | ● | ● |  |  |  |  |  | ● | ● | ● |  | ● | |  |  |
|  | LDP |  |  | ● | ● |  |  |  |  |  |  | ● | ● |  | ● | | ● | ● |
|  | PGB |  |  | ● | ● |  |  |  |  | ● |  | ● | ● |  | ● | | ● | ● |
|  | SJO |  |  | ● |  |  |  |  |  |  | ● | ● | ● |  | ● | | ● | ● |
| East | PCP |  |  | ● | ● |  |  |  |  | ● |  | ● | ● |  |  | | ● |  |
|  | PCD |  | ● | ● | ● |  |  |  |  | ● |  | ● |  |  | ● | |  |  |
|  | PNDL |  |  |  | ● |  |  |  |  |  | ● | ● | ● |  | ● | | ● |  |
|  | PBSB |  |  | ● | ● |  |  | ● |  | ● | ● |  |  | ● | ● | | ● | ● |
|  | PDA |  |  | ● | ● |  |  |  |  | ● | ● | ● | ● |  | ● | | ● |  |
|  | PBS |  |  |  | ● |  | ● |  |  | ● |  | ● |  | ● | ● | | ● |  |
| Center | PTC |  | ● | ● | ● |  |  |  |  |  |  | ● | ● |  |  | |  |  |
|  | PHY |  |  | ● | ● |  |  |  |  | ● |  | ● | ● |  | ● | |  |  |
|  | PDP |  | ● | ● | ● |  |  |  |  |  |  | ● | ● |  |  | |  |  |
|  | PSA |  |  | ● | ● |  |  |  |  | ● | ● |  | ● |  |  | | ● |  |
| Percentage of populations with null alleles | | 0.0 | 15.8 | **89.5** | **84.2** | 0.0 | 5.3 | 21.1 | 5.3 | 57.9 | 47.4 | **89.5** | **73.7** | 10.5 | **78.9** | | **73.7** | 21.1 |

Presence: dotted boxes; Absence: empty boxes. Note that loci showing null alleles in more than 70% of the populations (in bold: Aealbmic4, Aealbmic5, Aealbmic12, Aealbmic13, Albdi6, Albtri3) were excluded from the analyses.
