# Supplementary material for: High genetic diversity but no geographical structure of Aedes albopictus populations in Réunion Island
Source: Parasit Vectors. 2019 Dec 19;12:597. doi: 10.1186/s13071-019-3840-x (PMC6924041; doi:10.1186/s13071-019-3840-x)
Supplement: Supplementary file 4 — Additional file 4: Table S4. Microsatellite variation in the 19 Aedes albopictus populations. Abbreviations: n, number of individuals analyzed; Na, number of alleles; Ne, effective number of alleles; Np, number of private alleles; HO, observed heterozygosities; HE, expected heterozygosities; FIS, inbreeding coefficient (*P < 0.05, **P < 0.01, ***P < 0.001); SE, standard error. [file 13071_2019_3840_MOESM4_ESM.doc]

**Additional file 4**: **Table S4.** Microsatellite variation in the 19 *Aedes albopictus* populations. *Abbreviations*: *n*, number of individuals analyzed; *N_a_*, number of alleles; *N_e_*, effective number of alleles; *N_p_*, number of private alleles; *H_O_*, observed heterozygosities; *H_E_*, expected heterozygosities; *F_IS_*, inbreeding coefficient (**P* < 0.05, ***P* < 0.01, ****P* < 0.001); SE, standard error.

|  |  |  | Aealbmic | | | | | | | | |  |  |
| --- | --- | --- | --- | --- | --- | --- | --- | --- | --- | --- | --- | --- | --- |
| Region | Sites | Index | 2 | 3 | 6 | 7 | 8 | 9 | 10 | 11 | 16 | Albtri45 | All ± SE |
| West | W-PRO (*n*=25) | N_a_ | 2 | 12 | 4 | 4 | 5 | 7 | 4 | 8 | 7 | 6 | 5.90 ± 0.89 |
|  |  | N_e_ | 1.99 | 3.17 | 1.39 | 2.33 | 3.19 | 4.15 | 2.2 | 5.17 | 3.99 | 4.98 | 2.56 ± 0.41 |
|  |  | N_p_ | 0 | 0 | 0 | 0 | 0 | 1 | 0 | 0 | 1 | 0 | 2 |
|  |  | H_O_ | 0.36 | 0.6 | 0.32 | 0.6 | 0.72 | 0.76 | 0.44 | 0.72 | 0.68 | 0.72 | 0.59 ± 0.05 |
|  |  | H_E_ | 0.5 | 0.69 | 0.28 | 0.57 | 0.69 | 0.76 | 0.54 | 0.81 | 0.75 | 0.8 | 0.64 ± 0.05 |
|  |  | F_IS_ | 0.28 | 0.12 | -0.13 | -0.05 | -0.05 | 0 | 0.19 | 0.11 | 0.09 | 0.1 | 0.09 *** |
|  | W-LPO (*n*=27) | N_a_ | 2 | 10 | 6 | 5 | 6 | 6 | 4 | 6 | 5 | 6 | 5.60 ± 0.64 |
|  |  | N_e_ | 1.8 | 3.76 | 2.38 | 2.76 | 3.89 | 3.46 | 1.94 | 3.44 | 2.31 | 4.4 | 3.01 ± 0.28 |
|  |  | N_p_ | 0 | 0 | 0 | 0 | 1 | 0 | 0 | 0 | 0 | 0 | 1 |
|  |  | H_o_ | 0.3 | 0.7 | 0.67 | 0.48 | 0.48 | 0.93 | 0.37 | 0.48 | 0.52 | 0.89 | 0.58 ± 0.07 |
|  |  | H_E_ | 0.44 | 0.73 | 0.58 | 0.64 | 0.74 | 0.71 | 0.49 | 0.71 | 0.57 | 0.77 | 0.64 ± 0.04 |
|  |  | F_IS_ | 0.33 | 0.04 | -0.15 | 0.24 | 0.35 | -0.3 | 0.24 | 0.32 | 0.08 | -0.15 | 0.11 ** |
|  | W-ERM (*n*=27) | N_a_ | 2 | 10 | 6 | 6 | 4 | 5 | 4 | 9 | 5 | 6 | 5.70 ± 0.75 |
|  |  | N_e_ | 1.91 | 4.6 | 1.92 | 1.85 | 3.19 | 3.26 | 2.02 | 4.67 | 3.01 | 3.71 | 3.01 ± 0.34 |
|  |  | N_p_ | 0 | 1 | 0 | 0 | 0 | 0 | 0 | 1 | 0 | 0 | 2 |
|  |  | H_o_ | 0.33 | 0.7 | 0.52 | 0.48 | 0.59 | 0.78 | 0.26 | 0.67 | 0.7 | 0.74 | 0.58 ± 0.06 |
|  |  | H_E_ | 0.48 | 0.78 | 0.48 | 0.46 | 0.69 | 0.69 | 0.51 | 0.79 | 0.67 | 0.73 | 0.63 ± 0.04 |
|  |  | F_IS_ | 0.3 | 0.1 | -0.08 | -0.05 | 0.14 | -0.12 | 0.49 | 0.15 | -0.05 | -0.01 | 0.1 ** |

**Additional file 4**: **Table S4.** Continued

|  |  |  | Aealbmic | | | | | | | | |  |  |
| --- | --- | --- | --- | --- | --- | --- | --- | --- | --- | --- | --- | --- | --- |
| Region | Sites | Index | 2 | 3 | 6 | 7 | 8 | 9 | 10 | 11 | 16 | Albtri45 | All ± SE |
| West | W-P3B (*n*=29) | N_a_ | 2 | 9 | 3 | 5 | 4 | 5 | 3 | 7 | 3 | 6 | 4.70 ± 0.68 |
|  |  | N_e_ | 1.89 | 5.26 | 1.7 | 1.49 | 2.09 | 4 | 1.63 | 4.79 | 1.59 | 3.09 | 2.75 ± 0.45 |
|  |  | N_p_ | 0 | 0 | 0 | 0 | 0 | 0 | 0 | 0 | 0 | 0 | 0 |
|  |  | H_o_ | 0.48 | 0.79 | 0.38 | 0.34 | 0.34 | 0.59 | 0.21 | 0.52 | 0.38 | 0.79 | 0.48 ± 0.06 |
|  |  | H_E_ | 0.47 | 0.81 | 0.41 | 0.33 | 0.52 | 0.75 | 0.39 | 0.79 | 0.37 | 0.68 | 0.55 ± 0.06 |
|  |  | F_IS_ | -0.03 | 0.02 | 0.08 | -0.05 | 0.34 | 0.22 | 0.46 | 0.35 | -0.02 | -0.17 | 0.14 *** |
|  | W-ESL (*n*=30) | N_a_ | 2 | 11 | 4 | 3 | 4 | 5 | 5 | 9 | 6 | 5 | 5.40 ± 0.86 |
|  |  | N_e_ | 1.97 | 7.73 | 2.15 | 2.05 | 3.25 | 2.75 | 1.86 | 4.15 | 3.06 | 4.42 | 3.34 ± 0.56 |
|  |  | N_p_ | 0 | 0 | 0 | 0 | 0 | 0 | 0 | 0 | 0 | 0 | 0 |
|  |  | H_o_ | 0.47 | 0.93 | 0.5 | 0.5 | 0.53 | 0.6 | 0.33 | 0.6 | 0.67 | 0.67 | 0.58 ± 0.05 |
|  |  | H_E_ | 0.49 | 0.87 | 0.53 | 0.51 | 0.69 | 0.64 | 0.46 | 0.76 | 0.67 | 0.77 | 0.64 ± 0.04 |
|  |  | F_IS_ | 0.05 | -0.07 | 0.06 | 0.02 | 0.23 | 0.06 | 0.28 | 0.21 | 0.01 | 0.14 | 0.11 ** |
|  | W-PLA (*n*=30) | N_a_ | 2 | 11 | 4 | 3 | 6 | 5 | 5 | 7 | 5 | 5 | 5.30 ± 0.78 |
|  |  | N_e_ | 1.97 | 6.21 | 1.47 | 1.62 | 4.23 | 2.97 | 2.08 | 4.01 | 3.3 | 3.76 | 3.16 ± 0.46 |
|  |  | N_p_ | 0 | 0 | 0 | 0 | 0 | 0 | 0 | 0 | 0 | 0 | 0 |
|  |  | H_o_ | 0.67 | 0.77 | 0.27 | 0.37 | 0.77 | 0.73 | 0.4 | 0.5 | 0.77 | 0.7 | 0.59 ± 0.06 |
|  |  | H_E_ | 0.49 | 0.84 | 0.32 | 0.38 | 0.76 | 0.66 | 0.52 | 0.75 | 0.7 | 0.73 | 0.62 ± 0.06 |
|  |  | F_IS_ | -0.36 | 0.09 | 0.16 | 0.04 | 0 | -0.11 | 0.23 | 0.33 | -0.1 | 0.05 | 0.05 * |

**Additional file 4**: **Table S4.** Continued

|  |  |  | Aealbmic | | | | | | | | | |  | |  | |
| --- | --- | --- | --- | --- | --- | --- | --- | --- | --- | --- | --- | --- | --- | --- | --- | --- |
| Region | Sites | Index | 2 | 3 | 6 | 7 | 8 | 9 | 10 | 11 | 16 | Albtri45 | | All ± SE | |  |
| West | W-LDP (*n*=26) | N_a_ | 2 | 9 | 5 | 6 | 5 | 7 | 5 | 8 | 6 | 6 | | 5.90 ± 0.61 | |  |
|  |  | N_e_ | 1.6 | 3.81 | 2.47 | 2.04 | 3.6 | 3.99 | 2.36 | 3.72 | 2.73 | 3.94 | | 3.02 ± 0.28 | |  |
|  |  | N_p_ | 0 | 0 | 0 | 0 | 0 | 0 | 0 | 0 | 0 | 0 | | 0 | |  |
|  |  | H_o_ | 0.35 | 0.65 | 0.54 | 0.62 | 0.58 | 0.81 | 0.46 | 0.69 | 0.54 | 0.54 | | 0.58 ± 0.04 | |  |
|  |  | H_E_ | 0.38 | 0.74 | 0.59 | 0.51 | 0.72 | 0.75 | 0.58 | 0.73 | 0.63 | 0.75 | | 0.64 ± 0.04 | |  |
|  |  | F_IS_ | 0.08 | 0.11 | 0.09 | -0.21 | 0.2 | -0.08 | 0.2 | 0.05 | 0.15 | 0.28 | | 0.11 ** | |  |
|  | W-PGB (*n*=28) | N_a_ | 2 | 9 | 4 | 6 | 6 | 4 | 4 | 7 | 5 | 7 | | 5.40 ± 0.64 | |  |
|  |  | N_e_ | 2 | 5.3 | 2.27 | 1.97 | 4.87 | 2.09 | 2.8 | 3.87 | 3.66 | 4.13 | | 3.30 ± 0.39 | |  |
|  |  | N_p_ | 0 | 0 | 0 | 0 | 0 | 0 | 0 | 0 | 0 | 0 | | 0 | |  |
|  |  | H_o_ | 0.43 | 0.75 | 0.5 | 0.46 | 0.89 | 0.57 | 0.29 | 0.75 | 0.71 | 0.5 | | 0.59 ± 0.06 | |  |
|  |  | H_E_ | 0.5 | 0.81 | 0.56 | 0.49 | 0.79 | 0.52 | 0.64 | 0.74 | 0.73 | 0.76 | | 0.66 ± 0.04 | |  |
|  |  | F_IS_ | 0.14 | 0.08 | 0.11 | 0.06 | -0.12 | -0.09 | 0.56 | -0.01 | 0.02 | 0.34 | | 0.12 *** | |  |
|  | W-SJO (*n*=28) | N_a_ | 2 | 10 | 2 | 3 | 5 | 5 | 4 | 8 | 6 | 5 | | 5.00 ± 0.80 | |  |
|  |  | N_e_ | 1.85 | 4.98 | 1.32 | 1.81 | 4.6 | 2.93 | 2.51 | 4.93 | 2.93 | 3.42 | | 3.13 ± 0.42 | |  |
|  |  | N_p_ | 0 | 0 | 0 | 0 | 0 | 0 | 0 | 0 | 0 | 0 | | 0 | |  |
|  |  | H_o_ | 0.5 | 0.71 | 0.29 | 0.5 | 0.68 | 0.61 | 0.61 | 0.61 | 0.71 | 0.5 | | 0.57 ± 0.04 | |  |
|  |  | H_E_ | 0.46 | 0.8 | 0.24 | 0.45 | 0.78 | 0.66 | 0.6 | 0.8 | 0.66 | 0.71 | | 0.62 ± 0.06 | |  |
|  |  | F_IS_ | -0.09 | 0.11 | -0.17 | -0.12 | 0.13 | 0.08 | -0.01 | 0.24 | -0.08 | 0.29 | | 0.09 *** | |  |

**Additional file 4**: **Table S4.** Continued

|  |  |  | Aealbmic | | | | | | | | | |  | |  | |
| --- | --- | --- | --- | --- | --- | --- | --- | --- | --- | --- | --- | --- | --- | --- | --- | --- |
| Region | Sites | Index | 2 | 3 | 6 | 7 | 8 | 9 | 10 | 11 | 16 | Albtri45 | | All ± SE | |  |
| East | E-PCP (*n*=29) | N_a_ | 2 | 9 | 4 | 4 | 6 | 6 | 4 | 6 | 5 | 6 | | 5.20 ± 0.59 | |  |
|  |  | N_e_ | 1.92 | 5.08 | 2.56 | 2.04 | 4.5 | 3.84 | 2.2 | 4.27 | 2.5 | 4.31 | | 3.32 ± 0.38 | |  |
|  |  | N_p_ | 0 | 0 | 0 | 0 | 0 | 0 | 0 | 0 | 0 | 0 | | 0 | |  |
|  |  | H_o_ | 0.66 | 0.83 | 0.55 | 0.59 | 0.72 | 0.72 | 0.21 | 0.79 | 0.45 | 0.69 | | 0.62 ± 0.06 | |  |
|  |  | H_E_ | 0.48 | 0.8 | 0.61 | 0.51 | 0.78 | 0.74 | 0.55 | 0.77 | 0.6 | 0.77 | | 0.66 ± 0.04 | |  |
|  |  | F_IS_ | -0.37 | -0.03 | 0.09 | -0.15 | 0.07 | 0.02 | 0.62 | -0.04 | 0.25 | 0.1 | | 0.08 *** | |  |
|  | E-PCD (*n*=30) | N_a_ | 2 | 11 | 4 | 4 | 6 | 6 | 5 | 8 | 5 | 6 | | 5.70 ± 0.78 | |  |
|  |  | N_e_ | 1.72 | 6.55 | 1.87 | 1.8 | 4.26 | 4.83 | 2.83 | 4.63 | 3.06 | 4.7 | | 3.62 ± 0.51 | |  |
|  |  | N_p_ | 0 | 0 | 0 | 0 | 0 | 0 | 0 | 0 | 0 | 0 | | 0 | |  |
|  |  | H_o_ | 0.33 | 0.67 | 0.57 | 0.4 | 0.7 | 0.73 | 0.5 | 0.77 | 0.67 | 0.73 | | 0.61 ± 0.05 | |  |
|  |  | H_E_ | 0.42 | 0.85 | 0.47 | 0.45 | 0.77 | 0.79 | 0.65 | 0.78 | 0.67 | 0.79 | | 0.66 ± 0.05 | |  |
|  |  | F_IS_ | 0.21 | 0.21 | -0.22 | 0.1 | 0.08 | 0.07 | 0.23 | 0.02 | 0.01 | 0.07 | | 0.1 ** | |  |
|  | E-PNDL (*n*=27) | N_a_ | 2 | 13 | 3 | 6 | 5 | 5 | 4 | 7 | 6 | 6 | | 5.70 ± 0.94 | |  |
|  |  | N_e_ | 1.93 | 7.25 | 1.64 | 2.41 | 2.67 | 3.26 | 1.98 | 4.66 | 2.98 | 3.84 | | 3.26 ± 0.53 | |  |
|  |  | N_p_ | 0 | 0 | 0 | 0 | 0 | 0 | 0 | 0 | 0 | 0 | | 0 | |  |
|  |  | H_o_ | 0.44 | 0.93 | 0.41 | 0.67 | 0.67 | 0.59 | 0.52 | 0.56 | 0.7 | 0.74 | | 0.62 ± 0.05 | |  |
|  |  | H_E_ | 0.48 | 0.86 | 0.39 | 0.59 | 0.62 | 0.69 | 0.49 | 0.79 | 0.66 | 0.74 | | 0.63 ± 0.05 | |  |
|  |  | F_IS_ | 0.08 | -0.07 | -0.04 | -0.14 | -0.07 | 0.15 | -0.05 | 0.29 | -0.06 | 0 | | 0.03 | |  |

**Additional file 4**: **Table S4.** Continued

|  |  |  | Aealbmic | | | | | | | | | |  | |  | |
| --- | --- | --- | --- | --- | --- | --- | --- | --- | --- | --- | --- | --- | --- | --- | --- | --- |
| Region | Sites | Index | 2 | 3 | 6 | 7 | 8 | 9 | 10 | 11 | 16 | Albtri45 | | All ± SE | |  |
| East | E-PBSB (*n*=25) | N_a_ | 2 | 12 | 4 | 2 | 4 | 5 | 4 | 7 | 5 | 6 | | 5.10 ± 0.91 | |  |
|  |  | N_e_ | 1.89 | 6.38 | 1.52 | 1.04 | 3.21 | 3.29 | 2.59 | 4.33 | 2.1 | 4.9 | | 3.13 ± 0.53 | |  |
|  |  | N_p_ | 0 | 0 | 0 | 0 | 0 | 0 | 0 | 0 | 0 | 0 | | 0 | |  |
|  |  | H_o_ | 0.36 | 0.76 | 0.4 | 0.04 | 0.44 | 0.64 | 0.48 | 0.48 | 0.48 | 0.56 | | 0.47 ± 0.06 | |  |
|  |  | H_E_ | 0.47 | 0.84 | 0.34 | 0.04 | 0.69 | 0.7 | 0.61 | 0.77 | 0.52 | 0.8 | | 0.58 ± 0.08 | |  |
|  |  | F_IS_ | 0.24 | 0.1 | -0.17 | -0.02 | 0.36 | 0.08 | 0.22 | 0.38 | 0.08 | 0.3 | | 0.21 *** | |  |
|  | E-PDA (*n*=24) | N_a_ | 2 | 10 | 5 | 3 | 5 | 5 | 4 | 7 | 5 | 7 | | 5.30 ± 0.72 | |  |
|  |  | N_e_ | 1.95 | 7.84 | 2.32 | 1.71 | 4.4 | 3.06 | 2.43 | 3.99 | 2.88 | 4.55 | | 3.51 ± 0.58 | |  |
|  |  | N_p_ | 0 | 0 | 0 | 0 | 0 | 0 | 0 | 0 | 0 | 0 | | 0 | |  |
|  |  | H_o_ | 0.5 | 0.88 | 0.71 | 0.29 | 0.75 | 0.71 | 0.38 | 0.38 | 0.71 | 0.67 | | 0.60 ± 0.06 | |  |
|  |  | H_E_ | 0.49 | 0.87 | 0.57 | 0.41 | 0.77 | 0.67 | 0.59 | 0.75 | 0.65 | 0.78 | | 0.66 ± 0.05 | |  |
|  |  | F_IS_ | -0.03 | 0 | -0.25 | 0.3 | 0.03 | -0.05 | 0.36 | 0.5 | -0.09 | 0.15 | | 0.11 *** | |  |
|  | E-PBS (*n*=29) | N_a_ | 2 | 11 | 5 | 5 | 5 | 4 | 5 | 7 | 5 | 5 | | 5.40 ± 0.73 | |  |
|  |  | N_e_ | 1.94 | 6.7 | 1.83 | 2.89 | 4.09 | 2.96 | 2.92 | 2.45 | 2.32 | 4.71 | | 3.28 ± 0.48 | |  |
|  |  | N_p_ | 0 | 0 | 0 | 0 | 0 | 0 | 0 | 0 | 0 | 0 | | 0 | |  |
|  |  | H_o_ | 0.41 | 0.86 | 0.38 | 0.28 | 0.69 | 0.69 | 0.34 | 0.55 | 0.34 | 0.79 | | 0.53 ± 0.07 | |  |
|  |  | H_E_ | 0.49 | 0.85 | 0.45 | 0.65 | 0.76 | 0.66 | 0.66 | 0.59 | 0.57 | 0.79 | | 0.65 ± 0.04 | |  |
|  |  | F_IS_ | 0.15 | -0.01 | 0.16 | 0.58 | 0.09 | -0.04 | 0.48 | 0.07 | 0.39 | -0.01 | | 0.19 *** | |  |

**Additional file 4**: **Table S4.** Continued

|  |  |  | Aealbmic | | | | | | | | | |  | |  | |
| --- | --- | --- | --- | --- | --- | --- | --- | --- | --- | --- | --- | --- | --- | --- | --- | --- |
| Region | Sites | Index | 2 | 3 | 6 | 7 | 8 | 9 | 10 | 11 | 16 | Albtri45 | | All ± SE | |  |
| Center | C-PTC (*n*=28) | N_a_ | 2 | 11 | 4 | 5 | 6 | 3 | 5 | 7 | 6 | 6 | | 5.50 ± 0.78 | |  |
|  |  | N_e_ | 1.96 | 8.86 | 1.35 | 2.96 | 4.01 | 2.51 | 2.24 | 3.92 | 3.16 | 4.28 | | 3.52 ± 0.67 | |  |
|  |  | N_p_ | 0 | 0 | 0 | 0 | 0 | 0 | 0 | 0 | 0 | 0 | | 0 | |  |
|  |  | H_o_ | 0.5 | 0.75 | 0.29 | 0.64 | 0.75 | 0.54 | 0.61 | 0.64 | 0.61 | 0.82 | | 0.61 ± 0.05 | |  |
|  |  | H_E_ | 0.49 | 0.89 | 0.26 | 0.66 | 0.75 | 0.6 | 0.55 | 0.74 | 0.68 | 0.77 | | 0.64 ± 0.06 | |  |
|  |  | F_IS_ | -0.02 | 0.15 | -0.11 | 0.03 | 0 | 0.11 | -0.1 | 0.14 | 0.11 | -0.07 | | 0.06 ** | |  |
|  | C-PHY (*n*=29) | N_a_ | 2 | 10 | 4 | 5 | 6 | 5 | 5 | 8 | 4 | 5 | | 5.40 ± 0.70 | |  |
|  |  | N_e_ | 1.62 | 4.25 | 2.16 | 2.58 | 4.89 | 3.2 | 2.24 | 3.62 | 2.54 | 4.34 | | 3.14 ± 0.35 | |  |
|  |  | N_p_ | 0 | 0 | 0 | 0 | 1 | 0 | 0 | 0 | 0 | 0 | | 1 | |  |
|  |  | H_o_ | 0.31 | 0.79 | 0.59 | 0.59 | 0.69 | 0.72 | 0.38 | 0.83 | 0.59 | 0.76 | | 0.62 ± 0.05 | |  |
|  |  | H_E_ | 0.38 | 0.76 | 0.54 | 0.61 | 0.8 | 0.69 | 0.55 | 0.72 | 0.61 | 0.77 | | 0.64 ± 0.04 | |  |
|  |  | F_IS_ | 0.19 | -0.04 | -0.09 | 0.04 | 0.13 | -0.05 | 0.32 | -0.14 | 0.03 | 0.01 | | 0.05 | |  |
|  | C-PDP (*n*=30) | N_a_ | 2 | 9 | 3 | 4 | 6 | 5 | 5 | 8 | 6 | 5 | | 5.30 ± 0.67 | |  |
|  |  | N_e_ | 1.97 | 4.26 | 1.57 | 1.79 | 3.81 | 3.21 | 1.7 | 5.19 | 2.01 | 4.56 | | 3.00 ± 0.43 | |  |
|  |  | N_p_ | 0 | 0 | 0 | 0 | 0 | 0 | 0 | 0 | 0 | 0 | | 0 | |  |
|  |  | H_o_ | 0.53 | 0.43 | 0.37 | 0.47 | 0.77 | 0.8 | 0.37 | 0.7 | 0.5 | 0.8 | | 0.57 ± 0.06 | |  |
|  |  | H_E_ | 0.49 | 0.77 | 0.36 | 0.44 | 0.74 | 0.69 | 0.41 | 0.81 | 0.5 | 0.78 | | 0.60 ± 0.06 | |  |
|  |  | F_IS_ | -0.09 | 0.43 | -0.01 | -0.06 | -0.04 | -0.16 | 0.11 | 0.13 | 0.01 | -0.02 | | 0.06 *** | |  |

**Additional file 4**: **Table S4.** Continued

|  |  |  | Aealbmic | | | | | | | | |  |  |
| --- | --- | --- | --- | --- | --- | --- | --- | --- | --- | --- | --- | --- | --- |
| Region | Sites | Index | 2 | 3 | 6 | 7 | 8 | 9 | 10 | 11 | 16 | Albtri45 | All ± SE |
| Center | C-PSA (*n*=29) | N_a_ | 2 | 7 | 4 | 5 | 4 | 6 | 5 | 9 | 5 | 6 | 5.30 ± 0.60 |
|  |  | N_e_ | 1.53 | 4.7 | 1.8 | 2.12 | 3.09 | 3.5 | 2.9 | 5.61 | 2.18 | 3.88 | 3.13 ± 0.42 |
|  |  | N_p_ | 0 | 0 | 0 | 0 | 0 | 0 | 0 | 1 | 0 | 0 | 1 |
|  |  | H_o_ | 0.38 | 0.83 | 0.52 | 0.55 | 0.62 | 0.66 | 0.38 | 0.38 | 0.52 | 0.83 | 0.57 ± 0.05 |
|  |  | H_E_ | 0.35 | 0.79 | 0.44 | 0.53 | 0.68 | 0.71 | 0.65 | 0.82 | 0.54 | 0.74 | 0.63 ± 0.05 |
|  |  | F_IS_ | -0.09 | -0.05 | -0.17 | -0.05 | 0.08 | 0.08 | 0.42 | 0.54 | 0.04 | -0.12 | 0.11 *** |
