# Supplementary material for: High genetic diversity but no geographical structure of Aedes albopictus populations in Réunion Island
Source: Parasit Vectors. 2019 Dec 19;12:597. doi: 10.1186/s13071-019-3840-x (PMC6924041; doi:10.1186/s13071-019-3840-x)
Supplement: Supplementary file 5 — Additional file 5: Figure S1. Hierarchical clustering of Aedes albopictus individuals from the same population. Example showing the case of the population C-PSA (n = 25 individuals). a Dendrogram generated by the hierarchical clustering. b Principal component analysis. Abbreviation: Ind, individual. [file 13071_2019_3840_MOESM5_ESM.doc]

**
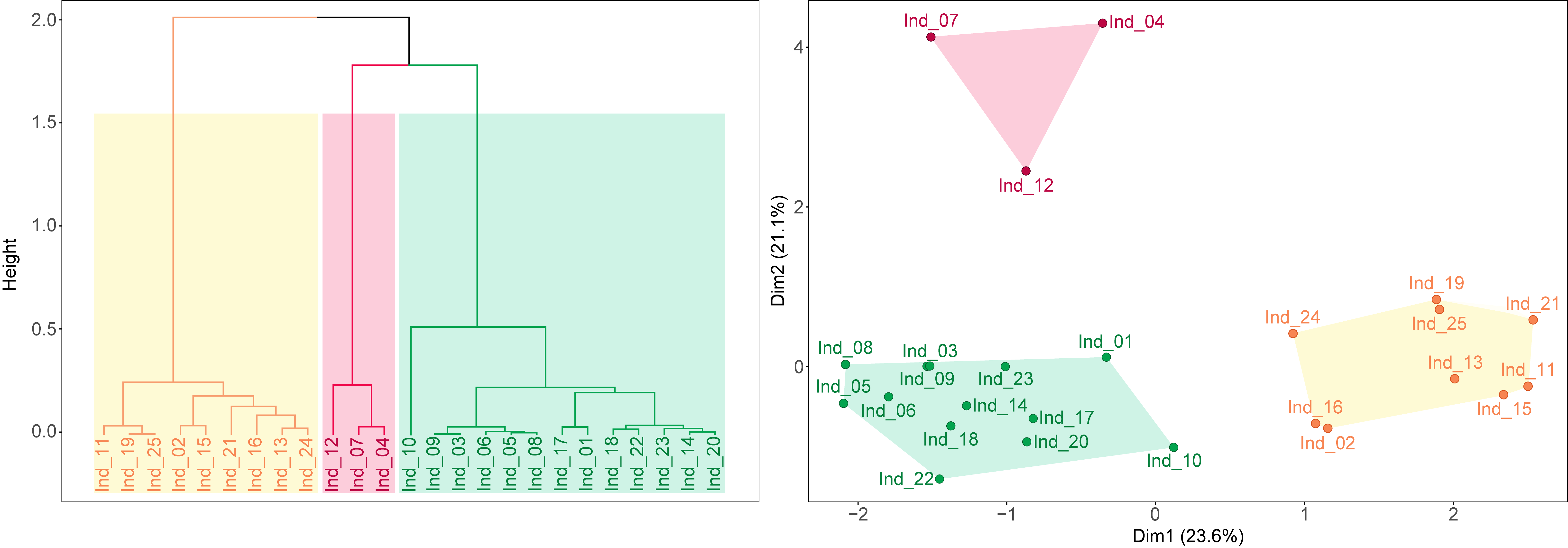
**

aaa

b

**Additional file 5: Figure S1.** Hierarchical clustering of Aedes albopictus individuals from the same population. Example showing the case of the population C-PSA (*n* = 25 individuals). **a** Dendrogram generated by the hierarchical clustering. **b** Principal component analysis. *Abbreviation*: Ind, individual.
