# Supplementary material for: High genetic diversity but no geographical structure of Aedes albopictus populations in Réunion Island
Source: Parasit Vectors. 2019 Dec 19;12:597. doi: 10.1186/s13071-019-3840-x (PMC6924041; doi:10.1186/s13071-019-3840-x)
Supplement: Supplementary file 6 — Additional file 6: Figure S2. Distribution of linkage disequilibrium Chi-square statistic estimated for each couple of loci. In grey: over the 100 subsampled datasets; in black: for the full dataset. [file 13071_2019_3840_MOESM6_ESM.doc]

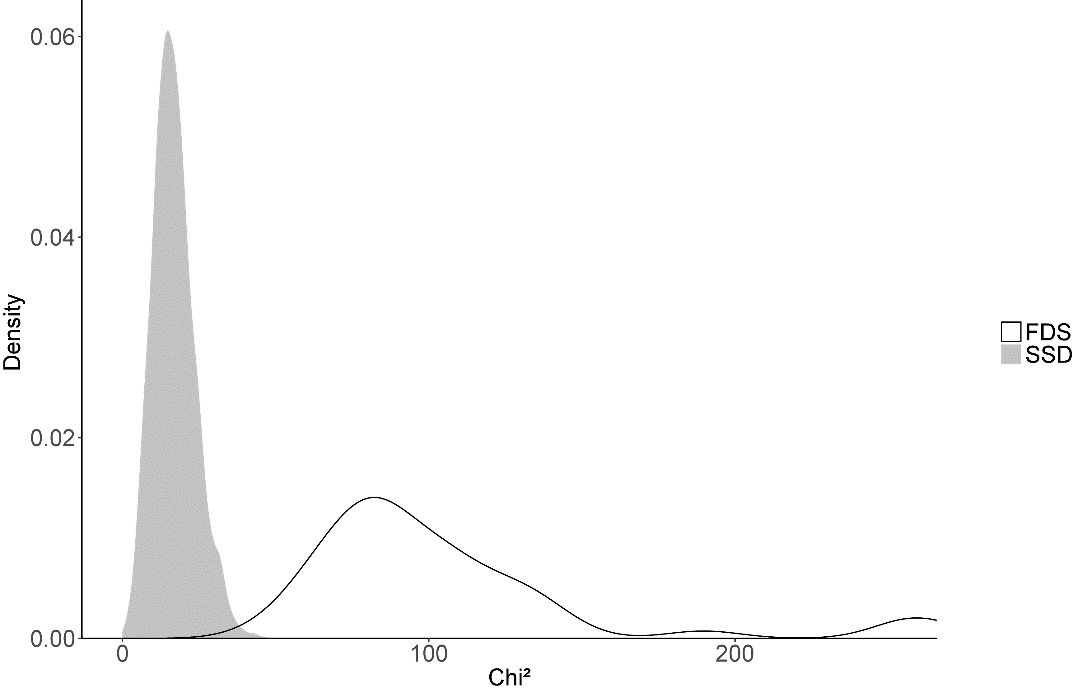


**Additional file 6: Figure S2**. Distribution of linkage disequilibrium Chi-square statistic estimated for each couple of loci. In grey: over the 100 subsampled datasets; in black: for the full dataset.
