# Supplementary material for: High genetic diversity but no geographical structure of Aedes albopictus populations in Réunion Island
Source: Parasit Vectors. 2019 Dec 19;12:597. doi: 10.1186/s13071-019-3840-x (PMC6924041; doi:10.1186/s13071-019-3840-x)
Supplement: Supplementary file 7 — Additional file 7: Figure S3. Relationship between FST values for Aedes albopictus pairs of populations. a Comparisons of FST estimated using R (x-axis) and GenAlEx v.6.5 [43] (y-axis) without taking into account relatives. b Comparisons of FST estimated using R without taking relatives into account (x-axis) and by taking relatives into account (y-axis). Full line: regression line; dotted line: y = x. [file 13071_2019_3840_MOESM7_ESM.doc]

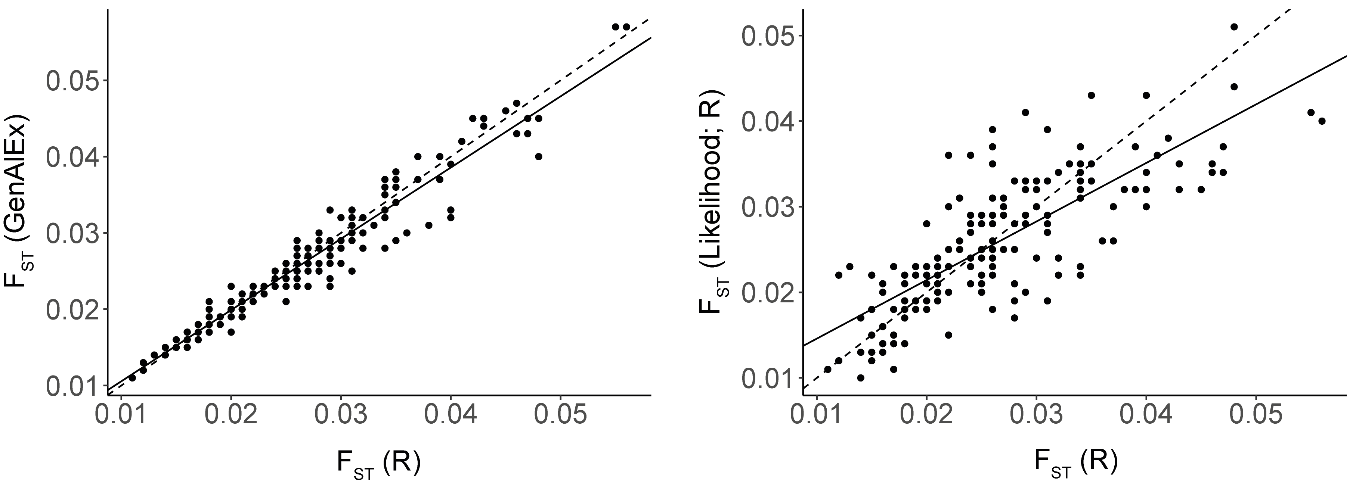


b

a

**Additional file 7: Figure S3**. Relationship between *F_ST_* values for *Aedes albopictus* pairs of populations. (a) comparisons of *F_ST_* estimated using R (eq. 1; x axis) and GenAlEx v.6.5 [3] (y axis) without taking into account relatives;
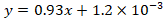
 (Pearson test: n = 171, t = 50.41; *R²* = 0.94; *P* < 10^-5^). (b) comparisons of *F_ST_* estimated using R without taking relatives into account (x axis) and by taking relatives into account (y axis);
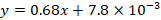
 (Pearson test: n = 171, t = 16.48; *R²* = 0.62; *P* < 10^-5^). Full line: regression line; dotted line: y = x.
