# Supplementary material for: High genetic diversity but no geographical structure of Aedes albopictus populations in Réunion Island
Source: Parasit Vectors. 2019 Dec 19;12:597. doi: 10.1186/s13071-019-3840-x (PMC6924041; doi:10.1186/s13071-019-3840-x)
Supplement: Supplementary file 8 — Additional file 8: Figure S4. Relationship between the FST and the distances by road calculated between pairs of mosquito populations. y = − 3.58 × 10−6 x + 2.61 × 10^−2^ (Pearsonʼs test: n = 171, t = − 0.15, R2 = 1.36 × 10^−4^, P = 0.88). [file 13071_2019_3840_MOESM8_ESM.doc]

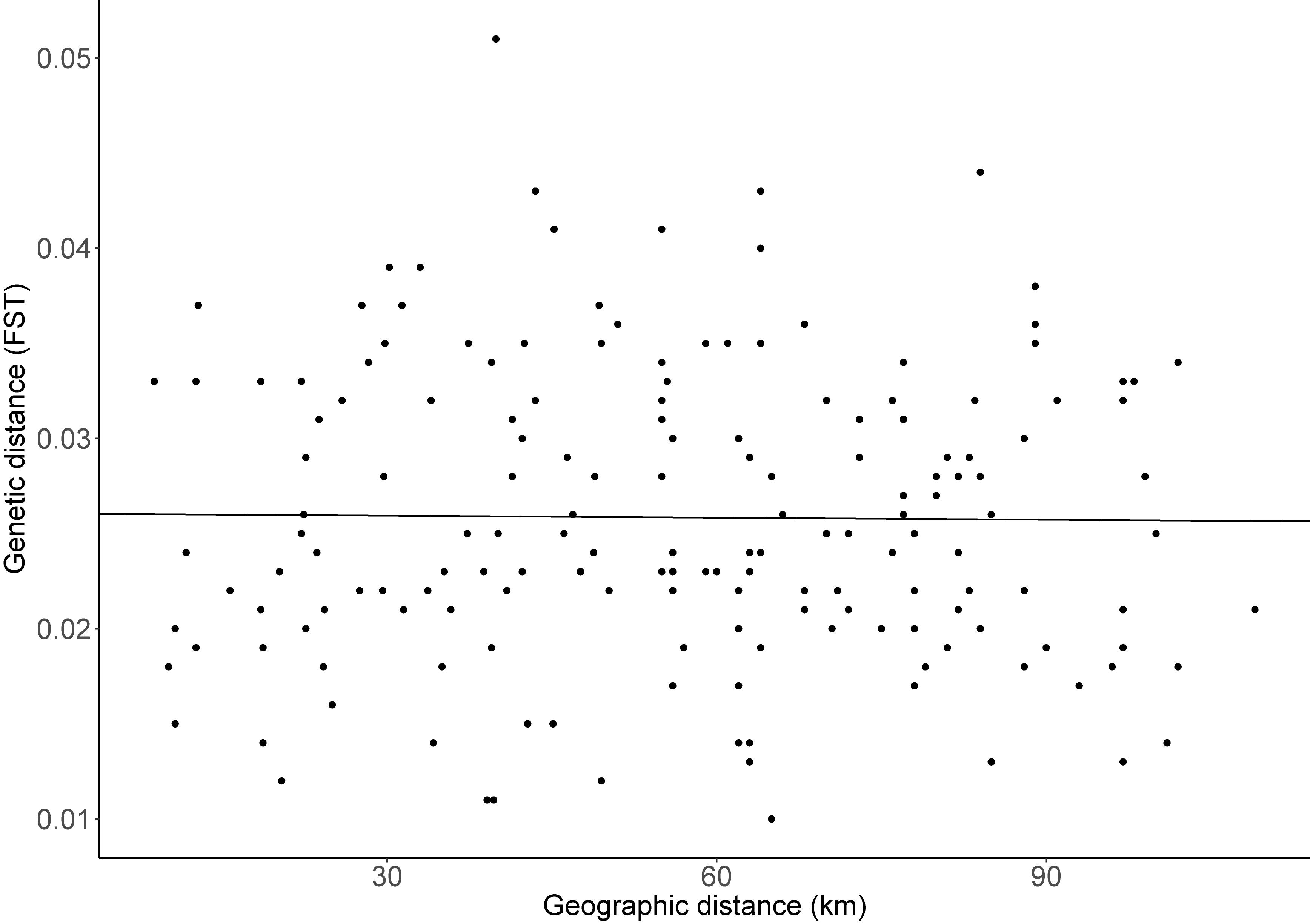


**Additional file 8: Figure S4**. Relationship between the *F_ST_* and the distances by road calculated between pairs of mosquito populations. *y* = -3.58 × 10^-6^ *x* + 2.61 × 10^-2^ (Pearson test: n = 171, t = -0.15, *R²* = 1.36x10^-4^, *P* = 0.88).
